# Vertebral Metastasis in a Bronchial Carcinoid: A Rare Case Report with More than 3-Year Follow-Up and Review of the Literature

**DOI:** 10.3390/diagnostics15091128

**Published:** 2025-04-28

**Authors:** Carlo Biz, Maria Grazia Rodà, Fabiana Mori, Lorenzo Costa, Joseph Domenico Gabrieli, Francesco Causin, Pietro Ruggieri

**Affiliations:** 1 Orthopedics and Orthopedic Oncology, Department of Surgery, Oncology and Gastroenterology (DiSCOG), University of Padova, Via Giustiniani 3, 35128 Padova, Italy; mariagrazia.roda@aopd.veneto.it (M.G.R.); fabianamori01@gmail.com (F.M.); lorenzcosta@outlook.it (L.C.); pietro.ruggieri@unipd.it (P.R.); 2 Neuroradiology Unit, Padua University Hospital, Via Giustiniani 2, 35128 Padova, Italy; josephdomenico.gabrieli@aopd.veneto.it (J.D.G.); francesco.causin@aopd.veneto.it (F.C.)

**Keywords:** lung tumour, neuroendocrine tumour, bronchial carcinoid, skeletal metastases, vertebroplasty

## Abstract

**Background:** Skeletal metastases from carcinoid tumours are extremely rare. Their correct diagnosis is a challenging problem for clinicians and pathologists, with important clinical implications for patients. In most cases, examination for the possible presence of skeletal metastases is initiated only when patients present symptoms suggestive of skeletal metastases. **Case presentation:** In this paper, the authors present the case of a middle-aged woman suffering from back pain due to a bronchial carcinoid that metastasised to the spine. We managed skeletal metastases with vertebroplasty and achieved excellent results and the complete remission of symptoms six months after the procedure. **Conclusions:** The relevance of this case report is that it highlights the importance of correctly diagnosing and treating these rare lesions to improve the quality of life of metastatic oncologic patients.

## 1. Introduction

Lung neuroendocrine tumours are a family of pathologies with common morphological, ultrastructural, immunohistochemical and molecular characteristics [1]. They are classified into four subtypes: small cell neuroendocrine carcinoma, large cell neuroendocrine carcinoma, typical carcinoid and atypical carcinoid [2]. Typical carcinoid tumours are less common than malignant tumours, representing 0.4–3% of all pulmonary neoplasms [3]. They usually occur in females younger than 60 years, with two peaks of incidence at 35 and 55 years [4,5]. The carcinoid tumour grows slowly in the central zone in 70% of cases. Its symptoms and signs occur in approximately two-thirds of patients, including cough, recurrent pneumonia and haemoptysis [6,7]. In contrast, patients with peripheral localised carcinoids are more often asymptomatic [7]. Paraneoplastic “carcinoid” syndrome, characterised by sudden skin flushing, diarrhoea, asthma-like crises and elevated levels of 5-hydroxy-indolacetic acid (5HIAA) in the urine, is uncommon in lung carcinoid patients (1% of cases) but can occur more frequently (5–10%) in patients with an atypical variant and/or in the presence of distant metastases [8].

CT scans of the chest and upper abdomen are the gold standard for carcinoid diagnosis, showing the typical hypervascularisation and identifying any lymph node or liver metastases [9]. Scintigraphy with radiolabelled octreotide is a valuable technique that highlights both the primary tumour and metastases [10]. However, only one-third of carcinoids express somatostatin receptors, and the accuracy of this method is limited by the presence of false positives (e.g., pneumonia, sarcoidosis and non-small cell lung cancer (NSCLC)) [11]. Positron emission tomography/computed tomography (PET-CT) does not appear to be more useful than CT since fluorodeoxyglucose (FDG) uptake by carcinoids is generally low [12].

Metastases in atypical carcinoid tumours occur late, often affecting lymph nodes, liver or bones, without a distinct radiological pattern [1]. Hence, its correct diagnosis is a challenging problem for clinicians and pathologists, with important clinical implications for patients. Since recurrences and distant metastases in CT can develop years after resection of the primary tumour, follow-up care for a minimum of 10 years is indicated [1]. Surgical treatment at the metastatic stage can be considered if the metastases are resectable [13]. Octreotide has become the main therapeutic regimen for treating carcinoid syndrome-related complaints [14].

The current literature on the management of metastatic lung neuroendocrine tumours remains scarce, and there is still uncertainty regarding optimal treatment strategies. As skeletal metastasis is now increasingly recognised, there is a need to improve the awareness of possible clinical approaches.

This paper aimed to share direct knowledge of the case of a 53-year-old woman with recurrent back pain due to metastatic typical bronchial carcinoid and to highlight the importance of correctly diagnosing and treating these rare lesions to improve the quality of life of these patients. To our knowledge, this is the first published report on this specific condition, which includes more than three years of follow-up after the initial orthopaedic treatment.

## 2. Case Presentation

**Figure 1:**
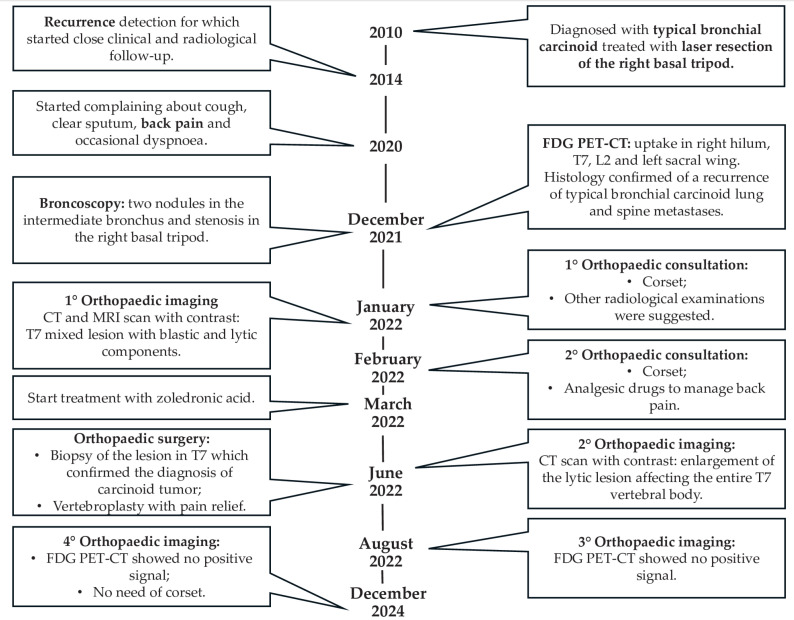
Flow chart of the case report.

The current case report was described in accordance with the Consensus-based Clinical Case Reporting Guideline as proposed in 2014 [15] and following the Flow Diagram for Case Reports as updated in 2017 (Figure 1) [16].

A 53-year-old woman with a history of seronegative spondyloarthritis, hypertension, marginal B-cell lymphoma and eye conditions was diagnosed with bronchial typical carcinoid in 2010. Initially, she underwent laser resection of the right basal tripod with stenotic results, but recurrence was detected in 2014 after transbronchial biopsy, with close clinical and radiological follow-up monitoring.

**Figure 2:**
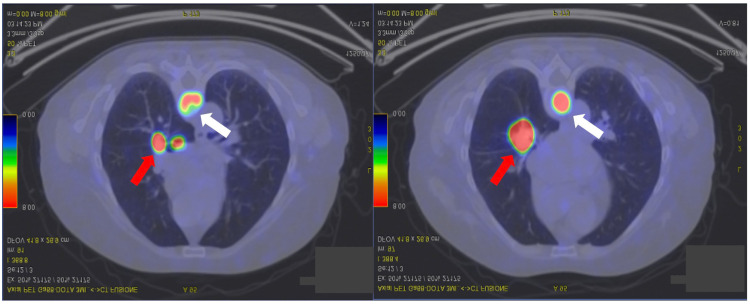
Two axial sections of the FDG CT-PET image showing uptake at the right lung hilum (red arrow) and at the vertebral spine on T7 (white arrow).

In 2020, the patient complained of cough, clear sputum and occasional dyspnoea. In December 2021, she underwent FDG PET-CT, which revealed FDG uptake in the right hilum [maximum standardised uptake (SUVmax) value 8] and in the T7 (SUVmax 12), L2 (SUVmax 3.3) and left sacral wing (SUVmax 2.4) levels in the spine (Figure 2).

**Figure 3:**
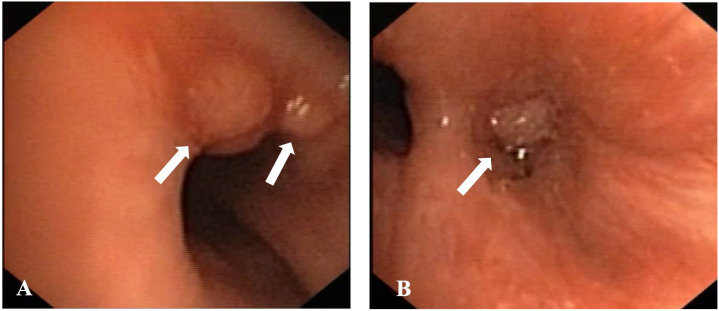
Endoscopic images obtained during bronchoscopy performed at our centre in Padova showing the presence of two nodules at the beginning of the intermediate bronchus (arrows) (**A**) and the lateral basal segment (arrow), which is completely unexplorable, downstream (**B**).

A new bronchoscopy revealed two nodules in the intermediate bronchus and stenosis in the right basal tripod (Figure 3).

Histology confirmed a recurrence of typical bronchial carcinoid lung and spine metastases. Following oncological evaluation, the patient started medical therapy with Lanreotide 120 mg and Everolimus.

**Figure 4:**
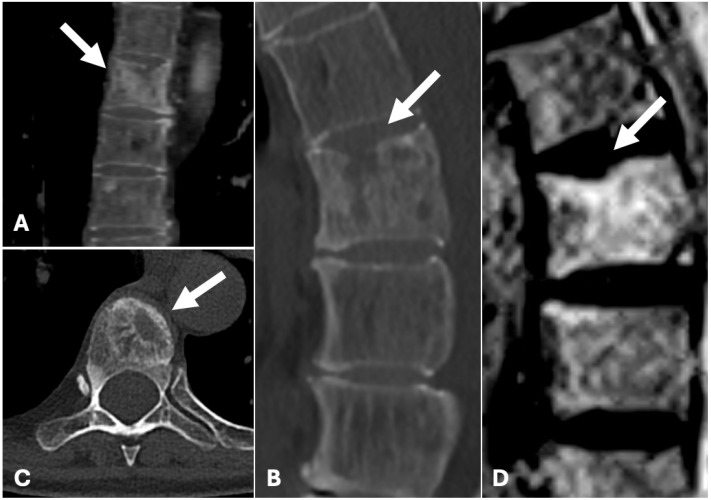
(**A**) Coronal, (**B**) sagittal and (**C**) axial CT reconstruction showing mixed lytic and blastic lesions of the T7 vertebral body (arrow). (**D**) MR image sagittal STIR image demonstrating high-intensity signals in the corresponding vertebral body.

By January 2022, due to consistent worsening back pain, the patient was led to undergo an orthopaedic clinical evaluation, where further radiological tests were suggested to better investigate the lesion. She was also instructed to wear an orthopaedic corset. Subsequently, a CT scan and an RMN with intravenous contrast medium were performed in January and February 2022, which showed a T7 mixed lesion with blastic and lytic components (Figure 4).

It was thus decided to manage the lesion with a three-month follow-up along with a prescription of an orthopaedic corset and analgesic drugs to manage back pain. In March 2022, the patient started therapy with zoledronic acid for the repair of the osseous metastases and to induce pain release. Due to the lack of pain control, a new CT scan with contrast medium was performed. It showed an enlargement of the lytic lesion affecting the entire vertebral body of T7. Therefore, it was decided to perform a biopsy of the lesion followed by vertebroplasty, which was executed in June 2022. The patient was positioned prone and the lesion was localised with XperCT. The biopsy was performed with a 13G needle, and the material was sent for a histologic examination. Using the same type of needle, polymethylmethacrylate was then injected into the vertebral body to fill the lesion (Figure 4).

**Figure 5:**
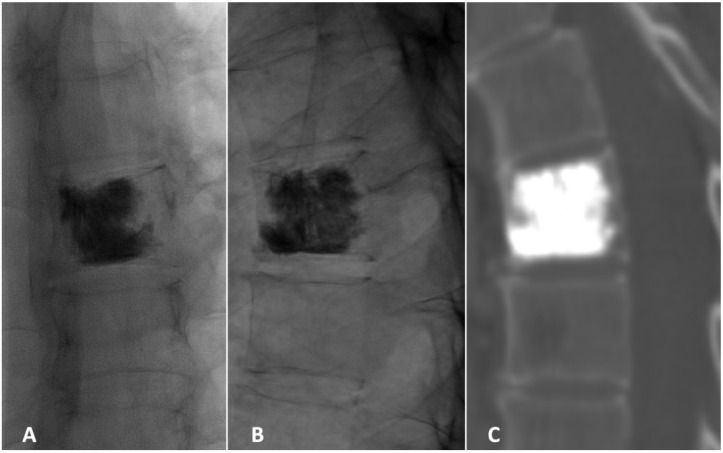
Frontal (**A**) and lateral (**B**) radiographs in the prone position immediately after vertebroplasty with 10 mL of polymethylmethacrylate (PMMA) was injected. The PMMA distributes into the lesion from the superior to the inferior endplate. (**C**) CT image showing sagittal reconstruction at the 6-month follow-up.

Histologic examination subsequently confirmed the diagnosis of carcinoid tumour localisation (cytokeratin MNF 116+, synaptophysin+, chromogranin+). After successful vertebroplasty, back pain regressed, and the follow-up FDG PET-CT scan performed on 16 August 2022 showed no positive signals. The most recent PET-CT scan in December 2024 confirmed no recurrence and the orthopaedic corset was no longer necessary. Figure 5 traces the clinical development from diagnosis to the last follow-up (Figure 5).

## 3. Literature Review

Only five cases of bone metastases from bronchial carcinoid tumours have been reported in the literature [17,18,19,20]. One of the first reported cases was a 48-year-old man who was diagnosed with bronchial carcinoids. At eight months after surgery, osteolytic metastasis was found in the proximal part of the left clavicle and multiple osteoblastic deposits in the right clavicle, vertebrae and sternum [17]. Another case was reported in a Spanish paper describing an 80-year-old man with pelvic localisation [18]. In February 2006, Ba D. Nguyen published an “Interesting Image” case of a 70-year-old man with a bronchial carcinoid and a lytic lesion of the left lateral mass of C5 treated with radiation therapy [20]. Finally, in a paper published in 2011, Takeshi Hori et al. reported two cases: the first involved a 59-year-old man with thoracic spine localisation treated with spinal decompression, while the second involved a 74-year-old man with an osteolytic lesion in the shaft of the left femur treated with intramedullary nail surgery [19]. Table 1 summarises the papers shown in this review (Table 1).

## 4. Discussion

The term carcinoid was first introduced by Oberndorfer in 1907 to describe a tumour of the small intestine that is clinically less aggressive than a carcinoma. The first report of a bronchial carcinoid tumour was by Laennac in 1831 [25]. Subsequently, tumours with similar clinical and biochemical features were found at other sites. A link has been established between the presence of these tumours and clinical syndromes due to various chemical substances produced by them [17].

Bronchial carcinoids constitute 4% of primary pulmonary neoplasms [21]. They were initially regarded as benign, but the occurrence of local and distant metastases made it clear that they were malignant [21]. Although skeletal metastases from carcinoid tumours were considered extremely rare in the past [22,23], they are now increasing due to longer survival and more advanced diagnostic techniques, reaching around 40% of all cases [24].

In most cases, examination for the presence of skeletal metastases was initiated in patients who already had clinical local pain symptoms that were suggestive of skeletal metastases [19]. However, patients with carcinoid tumours with skeletal metastasis do not always complain of local pain at the site of skeletal metastasis, as reported by Hory et al., or neurological pain [19]. The initial symptoms can range from minimal or no local pain to severe discomfort, as in our case. In some cases, bone metastasis may lead to pathological fractures, an outcome that should be avoided whenever possible. Other non-specific symptoms may include constipation, loss of appetite, nausea, frequent urination and confusion. However, skeletal metastasis may frequently remain undetected, as some patients do not complain of any symptoms [26].

Bone metastases from bronchial carcinoids are usually osteoblastic, which are in contrast to metastases from lung carcinomas, which are generally osteolytic [13,27]. Nevertheless, the literature review showed that osteoclastic metastases occur in fewer cases, suggesting once more that this is a very complex disease [17]. Furthermore, this characteristic discovery of a spinal osteolytic lesion and bone destruction should not only suggest the presence of a metastasis but it could also conceal a primary bone tumour, such as a giant cell tumour. Hence, a bone biopsy is mandatory for a proper diagnosis and the following treatment (conservative, adjuvant, surgical or a combination of these therapies), which can offer pain relief and a better prognosis [28].

The mechanisms of the metastases in bronchial carcinoid tumours are not yet fully understood, mainly due to their rarity and typically subtle behaviour. However, they are believed to alter vascular permeability, inducing cytoskeleton rearrangement and directional cell migration and reawakening dormant cancer cells [29]. This duality of the osteoblastic or osteoclastic metastases reflects the peculiar biological behaviour of a bronchial carcinoid. It may also be influenced by tumour grade, hormonal activity and individual patient variability. Hence, the metastatic spread occurs via haematogenous routes due to the tumour’s rich vascularisation, in particular from the venous system, and is neuroendocrinal in origin [29]. An indolent clinical course generally characterises metastases from this type of tumour. Still, they may sometimes be found in regional lymph nodes or distant sites like the liver, brain, subcutaneous tissue, skin, mammary glands, eyes and adrenals. Moreover, they can occur late, even decades, after primary tumour surgery. Therefore, it is essential to choose the correct diagnostic imaging technique. While bone and octreotide scintigraphy offer acceptable sensitivity, MRI provides higher sensitivity in detecting bone metastases [30]. FDG PET-CT is also valuable for identifying multiple bone metastases from carcinoid tumours [30]. Moreover, while the CT and MRI literature is limited, bone scintigraphy is a sensitive and reliable method but is typically used when symptoms such as back pain appear [26]. In the present case report, it was decided to adopt FDG PET-CT, which successfully revealed spine metastases.

Advances in the molecular understanding of these tumours have enabled earlier detection of bone metastases through targeted agents like 111In-pentetreotide and 131I-MIBG [31]. Lastly, laboratory data can help identify bone metastases, such as alkaline phosphatase, which can indicate bone disruption, and calcium, which can indicate bone damage. These tests can help identify metastases, but imaging techniques are essential for a correct and timely diagnosis [32].

**Figure 6:**
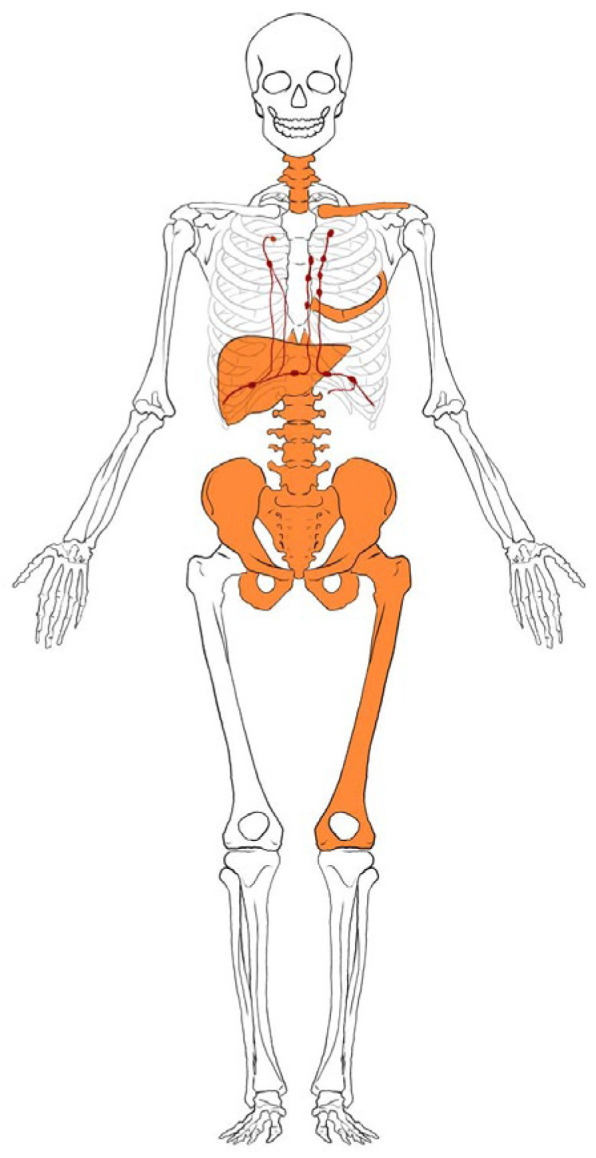
The most common sites of metastasis are highlighted in orange.

The cases reported in the literature illustrate the diverse clinical presentations and disease progression patterns, with metastases appearing months or years after the initial tumour diagnosis, as corroborated by our case [17,18,19]. Therefore, long-term follow-up should be considered mandatory for patients with bronchial carcinoids. In addition, although the spine seems the most common target, the variability in metastatic sites, including the rib, clavicle, pelvis, femur and liver, suggests that the disease can follow an unpredictable course, making continuous follow-up of multiple locations a crucial aspect of patient management (Figure 6) [17,18,19].

Focusing on spine metastases, 40% of cases present metastases to thoracic vertebrae (as in the current case report), 34% to the lumbar vertebrae and 32% to the cervical vertebrae [19]. Currently, there is no universally accepted approach to managing metastatic bronchial carcinoids, leading to heterogeneity in treatment strategies. While surgical resection of isolated metastases has been reported, other cases have been managed with radiotherapy or intramedullary stabilisation in the presence of skeletal involvement [17,18,19]. The patient in this case report, who was initially diagnosed with bronchial carcinoid in 2010, experienced a recurrence of her primitive tumour and developed vertebral metastases years later, emphasising once more the persistent nature of these tumours [32]. This case report, therefore, highlights the importance of strict follow-up and timely intervention in patients with carcinoid tumours. Despite advancements in imaging and therapy, the patient’s metastases were not fully characterised until back symptoms demanded further investigation [33,34]. Therefore, a multidisciplinary approach, involving the radiologist, oncologist, anatomopathologist and orthopaedist, is essential to manage the disease properly. Moreover, biopsy and vertebroplasty were performed consequentially in the same surgery, demonstrating a successful integrated approach to diagnosis and treatment, which appeared to be an effective treatment for reducing back pain. However, other studies have reported positive outcomes with radiotherapy and vertebral decompression, highlighting both a lack of consensus and a variety of indications based on the type and location of the metastasis [17,20]. Notably, these studies did not follow up with patients after surgery, leaving their long-term outcomes unknown [17,20]. In contrast, our study demonstrated that vertebroplasty also gave favourable results after almost three years of follow-up (34 months). Therefore, the multidisciplinary approach, involving orthopaedic intervention, medical therapy with Lanreotide and Everolimus, and vertebroplasty, was excellent care and management, significantly reducing her symptoms and improving her quality of life.

This case shows the necessity for long-term follow-up in carcinoid patients, as recurrences and distant metastases may manifest years after initial treatment, such as 10 years after, as in our case. It also highlights the value of personalised, multimodal treatment strategies in addressing both systemic and localised disease manifestations. Nevertheless, little data are available and further research is needed to establish optimal protocols for managing skeletal metastases in carcinoid patients.

## 5. Conclusions

In conclusion, this case contributes to the limited literature on skeletal metastases from bronchial carcinoid tumours. It emphasises the need for comprehensive diagnostic approaches, personalised and multidisciplinary therapeutic interventions and long-term follow-up to improve the prognosis and quality of life of these patients.

## Figures and Tables

**Table 1:** Summary of the papers with documented metastases from bronchial carcinoid tumour.

	n. pts	Sex	Ag	Metastasis Location	Symptoms Prior Surgery	Type of Intervention	Post-op Follow-Up	Symptoms After Surgery
**Ashraf MH (1977)** **[21]**	1	M	48	Left clavicle, multiple osteoblastic deposits in the right clavicle, vertebrae and sternum.	Pain at the right clavicle.	None.	-	-
**Pérez Angel F (2009)** **[22]**	1	M	80	Pelvic localisation.	-	-	-	-
**Hori T (2012)** **[23]**	2	M ^1/2^	59 *^1^ 74 *^2^	Thoracic spine localisation *^1^; osteolytic lesion in the shaft of the left femur *^2^.	Back pain and numbness in the lower limbs; none until lesion of the femur *^2^.	Spinal decompression *^1^; intramedullary nail and courettage *^2^.	1 y *^1^; -	Slight numbness in his leg *^1^. -
**Nguyen Ba D.** **(2006)** **[24]**	1	M	70	C5.	Not reported.	Radiation therapy.	-	-

(*) Only relating to ^1^: 59-year-old male patient; ^2^: 74-year-old male patient.

## Data Availability

The dataset supporting the conclusions of this review is available upon request to the corresponding author.
